# Supplementary material for: Intensity of Maternal Anxiety and Depressive Symptoms in Pregnancy Is Associated with Infant Emotional Regulation Problems
Source: Int J Environ Res Public Health. 2022 Nov 26;19(23):15761. doi: 10.3390/ijerph192315761 (PMC9741014; doi:10.3390/ijerph192315761)
Supplement: Supplementary file 1 [file ijerph-19-15761-s001.zip › ijerph-1974523-supplementary.pdf]

Table S1. Directionality of prenatal maternal symptoms and SES as predictors of infant crying and negative affectivity

	Infant Crying		*Negative Affectivity*	
	β	p	β	p
Step 1:				
Gender	-0.09	0.39	0.10	0.33
First Born			**-0.34**	**0.001**
Directionality of maternal postnatal symptoms	-0.10	0.37	0.10	0.32
	$R^2 = 0.02$		$R^2 = 0.12$	
Step 2:				
Directionality of maternal antenatal symptoms	-0.15	0.20	0.01	0.95
SES	0.03	0.79	0.19	0.06
	$\Delta R^2 = 0.02$		$\Delta R^2 = 0.03$	
Step 3:				
Directionality of maternal antenatal symptoms	-0.14	0.22	-0.03	0.74
SES	0.03	0.78	0.17	0.08
Directionality of maternal antenatal symptoms X SES	0.03	0.80	-0.19	0.07
	$\Delta R^2 = 0.001$		$\Delta R^2 = 0.03$	

Bold values indicate significant p< .05 results

SES: Socio Economic Status
